# Baltimore Dental College Commencement

**Published:** 1859-04

**Authors:** 


### EDITORIAL DEPARTMENT.

*Baltimore Dental College Commencement.*—The nineteenth annual commencement of the above named institution, was held Tuesday, March 1st, 1859, in the Saloon of the College Building. The exercises of the occasion were opened with prayer, by the Rev. Dr. James D. McCabe.

The names of the following Graduates, and Subjects of Theses, were announced by Prof. P. H. Austen, Dean of the Faculty.

*Graduates.*

| NAMES. | RESIDENCES. | SUBJECTS OF THESES. |
|---|---|---|
| JOSEPH WILLIAM BLANDY, | Maryland, | The Maxillary Antrum. |
| NATHAN DAVIS, | Pennsylvania, | Dental Hygiene. |
| WILLIAM DE HART, | Kentucky, | Mechanical Dentistry. |
| SAMUEL McMASTER FIELD, | Virginia, | The Blood. |
| ERVIN FLOYD, | S. Carolina, | Caries of the Teeth. |
| GEORGE ARCHER FRIERSON, | Louisiana, | Dental Education. |
| ALEXANDRE GARNIER, | Porto Rico, | Carie de Dents. |
| BENJAMIN FRANKLIN HOOPES, | Maryland, | Microscopic and Comparative Anatomy of the Teeth. |
| FRANCIS NAPOLEON KITCHELL, | Alabama, | Caries of the Teeth. |
| CHARLES KNOWER, | Missouri, | The Blood. |
| JAMES SAMFORD LAVENDER, | Georgia, | Diseases of the Gums. |
| ALEXANDER LAW, | Scotland, | Extraction of Teeth. |
| JOHN LOWRY McGEE, | Virginia, | Antrum Highmorianum. |
| ALBERT PHILLIPS, | Missouri, | Filling Teeth. |
| THEODORE PHILLIPS, | Louisiana, | Neuralgia. |
| EDWIN FAUST PIERCE, | Alabama, | The Maxillary Sinus. |
| SAMUEL SEVIER, | Alabama, | Mechanical Dentistry. |
| SAMUEL GREEN TODD, | Maryland, | Dental Caries. |
| SAMUEL YOUNG WEBB, | Alabama, | Alveolar Abscess. |
| SAMUEL WELCHENS, | Pennsylvania, | Surgery of the Mouth and Face. |

The degrees were conferred, and the diplomas delivered to each Graduate, by the President of the Faculty, Prof. C. A. Harris, who after this part of the ceremony had been concluded, delivered the valedictory address. At the conclusion of the exercises, a benediction was pronounced by the Rev. Dr. Roberts.

